# The Association Between Preoperative Insulin-Like Growth Factor 1 Levels and the Total Body Weight Loss in Women Post Laparoscopic Sleeve Gastrectomy

**DOI:** 10.1007/s11695-024-07077-9

**Published:** 2024-01-29

**Authors:** Mohamed Hamdy Khattab, Sami M. Said, Mina abdelmalak Fayez, Menatallah Mohamed Elaguizy, Abdelkarem A. A. Mohamed, Ahmed Mostafa Ghobashy

**Affiliations:** 1 Department of General Surgery, Faculty of Medicine, Cairo University, Cairo, Egypt; 2 Department of Clinical and Chemical Pathology, Faculty of Medicine, Cairo University, Cairo, Egypt

**Keywords:** Sleeve gastrectomy, Optimal initial clinical response, Metabolic profile, The insulin-like growth factor-1

## Abstract

**Background:**

Despite the well-described optimal initial clinical response of sleeve gastrectomy (SG) in the treatment of obesity, some patients do not achieve optimal initial clinical response. Insulin-like growth factor-1 (IGF-1) has currently shown an association with post-bariatric surgery weight loss. This study aimed to assess the IGF-1 levels in female patients with obesity, the change after surgery, and their association with the metabolic profile and weight loss after surgery.

**Patients and methods:**

This was a prospective study that was conducted on adult female patients who were recruited for SG. The patients underwent clinical and laboratory investigations that included the IGF-1 measurement. At the 1-year follow-up, the same clinical and laboratory measures were repeated.

**Results:**

This study included 100 female patients. At the 1-year follow-up, there was a statistically significant reduction in body mass index (BMI) (*p* < 0.001), fasting HbA1C levels (*p* < 0.001), and triglycerides (*p* < 0.001), as well as a statistically significant increase in HDL (*p* < 0.001) and IGF-1 (*p* < 0.001). Multiple regression analysis revealed that, among the patients baseline characteristics, the significant predictors for the percentage of total weight loss (%TWL) were the patients’ BMI (*p* < 0.001) and IGF-1 levels (*p* < 0.001). The ROC curve showed that an IGF1 cutoff value of ≤ 23 ng/ml detected suboptimal initial clinical response, with a sensitivity of 95.35% and a specificity of 100%.

**Conclusion:**

This study underscores the significant impact of SG on weight loss and metabolic improvements in female patients. Baseline IGF-1 levels emerged as a crucial predictor of optimal initial clinical response.

**Graphical Abstract:**

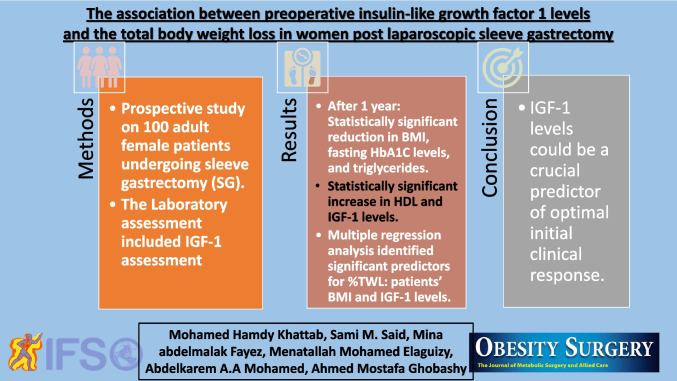

## Introduction

Obesity is associated with several health disorders, resulting in a reduction in the quality of life and overall life expectancy [1, 2]. Achieving weight loss reduces the morbidity and mortality of patients with obesity [3]. Surgical treatment of obesity remains a reliable solution for patients with severe obesity to reduce their weight and improve the associated medical disorders [4].

This has resulted in a continuously growing number of bariatric surgeries performed annually all over the world [5]. Being a simple technique with promising safety and efficacy [6], sleeve gastrectomy (SG) is currently the most commonly performed bariatric surgery [5].

Despite the well-described optimal initial clinical response of SG in the treatment of obesity, some patients do not achieve optimal initial clinical response after surgery and require revisional bariatric surgery [7]. The outcome of SG has been described as being associated with various factors, including the patients’ age, sex, body mass index (BMI), and obesity complications [8–14].

Among the factors described, insulin-like growth factor-1 (IGF-1) is currently linked to post-bariatric surgery weight loss [13]. IGF-1 is one of the anabolic hormones that enhance energy expenditure [15, 16]. In addition, there is an intricate association between IGF-1 and growth hormone (GH), with the latter inducing the hepatic synthesis and secretion of the former [17], which subsequently reflect the GH secretion status [18] and mediate its lipolytic effects [19, 20]. Therefore, it could be proposed to be associated with weight loss following surgery.

However, data regarding the IGF-1 levels in patients with obesity and their change after surgery show conflicting results. Also, there remains scarce evidence regarding its potential effect on post-LSG weight loss.

The present study aimed to assess the IGF-1 levels in female patients with obesity, the change after surgery, and the association between these levels, the patient’s metabolic profile, and weight loss after surgery.

## Patients and Methods

This was a prospective clinical study that was conducted on consecutive patients scheduled for LSG at our hospital during the period from December 2021 to December 2022 after obtaining Research Ethics Committee approval. The study followed the Helsinki Declaration.

After a multidisciplinary evaluation, patients’ eligibility for bariatric surgery was determined using criteria inspired by international societies concerned with obesity surgery [21–23]. Adult female patients who were recruited for LSG based on their choice after a thorough discussion with the surgeon, who presented the available choices, were included in the study. Patients with previous bariatric surgery and those who did not complete the follow-up visits were excluded from the study. The included patients provided informed written consent before being enrolled in the study.

The included patients underwent clinical assessment, including the anthropometric measures assessment and laboratory investigations that included the measurement of fasting serum glucose, glycosylated hemoglobin (HbA1c), lipid profile, and IGF-1, which was assessed with the Human Insulin-like Growth Factors 1, IGF-1ELISA Kit, SunLong Biotech Co., LTD, using the Elisa Plate Reader Statfax Chromate 4300 (U.S.A.). According to the manufacturer, normal IGF-1 levels range from 1.6 to 70 ng/mL.

The diagnostic criteria for diabetes are based on the measurement of blood glucose levels. According to the American Heart Association, a fasting level of 126 mg/dL or higher after an overnight fast of at least 8 h indicating diabetes mellitus [24]. The diagnostic criteria for hypertension are based on the measurement of blood pressure. According to the American Heart Association, a diagnosis of hypertension can be made in the following ways, a systolic blood pressure (SBP) of 130 mm Hg or higher or a diastolic blood pressure (DBP) of 80 mm Hg or higher on two or more readings taken on two or more occasions should diagnose hypertension [25]. The diagnostic criteria for dyslipidemia are based on the established guidelines, a diagnosis of dyslipidemia can be made when a total cholesterol level of 240 mg/dL or higher, a low-density lipoprotein (LDL) cholesterol level of 160 mg/dL or higher, and a high-density lipoprotein (HDL) cholesterol level of less than 40 mg/dL in men or less than 50 mg/dL in women [26].

Laparoscopic sleeve gastrectomy was performed as standardized [27]. In summary, the patients underwent routine preparation preoperatively, and the operation was conducted under general anesthesia. The required incisions were performed, and trocars were inserted. After the induction of pneumoperitoneum, the sleeved stomach pouch was created using a 36-Fr bougie. The stomach was resected starting from about 3–4 cm before the pyloric canal to the angle of His. Hemostasis was ensured throughout the surgery, and the leak testing was done. The patients received routine postoperative care and a schedule for postoperative visits.

At the 1-year follow-up, the same clinical and laboratory measures taken preoperatively were repeated. The total weight loss percentage (%TWL) was calculated [28]. A TWL percentage of less than 20% at the 1-year follow-up was considered suboptimal initial clinical response [29]. The resolution of the associated medical disorders was considered based on the American Society for Metabolic and Bariatric Surgery (ASMBS) recommendations. Remission of diabetes was considered if the HbA1c was less than 6 and fasting serum glucose was less than 100 mg/dL) in the absence of antidiabetic medications. Remission of hypertension was being normotensive (SBP < 120 mmHg and DBP < 80 mmHg). Remission of dyslipidemia was having LDL levels lower than < 100 mg/dL, triglycerides levels < 150 mg/dL, and HDL levels higher than 40 mg/dL [30].

### Study Outcomes

The outcomes of the current work were the potential association of IGF-1 with the patients’ weight status and the predictors of weight loss after surgery. The secondary outcomes were the short-term outcomes of LSG.

### Statistical Analysis

The patients’ data were analyzed using version 28 of the statistical software (SPSS, IBM Corp., Armonk, NY, USA). The patients’ data were expressed as a number and percentage if categorical, or a mean and standard deviation if numerical. An independent t test and a paired* t* test were used for the comparison of the numerical data, as appropriate. The McNemar’s test was used for the paired comparison of the categorical data. Pearson correlation analysis was used to test the correlation between numerical variables. Investigating the dynamic changes in IGF-1, the ∆ IGF (change from baseline) was calculated and its correlation with both %TWL and the change in Body Mass Index (∆ BMI) was explored. Multiple regression analysis was performed to assess the predictors for 1-year postoperative %TWL. The ROC curve was used to obtain the optimum IGF-1 cutoff value for the prediction of suboptimal initial clinical response. The level of significance was considered at a *p*-value of ≤ 0.05.

## Results

One hundred female patients who completed the follow-up period were included in this study. Their mean age was 36.98 ± 10.05 years. The mean baseline weight was 136.12 ± 22.86 kg, the mean baseline BMI was 49.68 ± 8.13 kg/m2, and the mean baseline waist circumference (WC) was 111.64 ± 22.47 cm (Table 1). The patients’ obesity complications and baseline laboratory findings are presented in Table 1.

**Table 1:** The patients’ characteristics before and 1-year after surgery (*n* = 100)

	Preoperative		1-year after surgery		*p*-value
	Mean ± SD	Range	Mean ± SD	Range	
Age (year)	36.98 ± 10.05	20—60			
Weight (Kg)	136.12 ± 22.86	94–210	90.1 ± 19.3	60–150	< 0.001*
BMI (Kg/m^2^)	49.68 ± 8.13	36.7–82	32.83 ± 6.76	21–58	< 0.001*
Waist circumference (cm)	111.64 ± 22.47	72–160	89.3 ± 21.71	43–136	< 0.001*
Fasting glucose (mg/dl)	124.76 ± 49.49	67–330	99.54 ± 25.23	68–222	< 0.001*
HBA1C (%)	6.34 ± 1.65	4.1–12	5.25 ± 0.79	4–7.2	< 0.001*
IGF-1 (ng/ml)	41.34 ± 11.72	18–60	108.47 ± 35.16	36–165	< 0.001*
Triglycerides (mg/dl)	144.16 ± 56.77	58–307	111.07 ± 45.15	48–269	< 0.001*
HDL (mg/dl)	43.39 ± 10.59	18–72	54.37 ± 10.53	36–90	< 0.001*
LDL (mg/dl)	124.51 ± 44.68	46–311	119.04 ± 35.43	52–205	0.34*
	Count	%	Count	%	
Obesity associated medical disorders					
Dyslipidemia	56	56%	9	9%	< 0.001*
Hypertension	41	41%	13	13%	< 0.001*
Type 2 diabetes mellitus	32	32%	17	17%	0.021*

At baseline, the IGF-1 levels showed a significant negative correlation with the patients’ age (*p* < 0.001), WC (*p* = 0.023), fasting serum glucose levels (*p* < 0.001), and fasting serum HbA1C levels (*p* < 0.001) (Table 2).

**Table 2:** Correlation between the baseline IGF-1 levels and the other baseline parameters

	Baseline IGF-1 levels	
	*r*	*p* value
Age	-0.353	< 0.001*
Baseline Weight	-0.12	0.235
Baseline BMI	-0.136	0.178
Baseline waist circumstance	-0.227	0.023*
Baseline fasting glucose	-0.356	< 0.001*
Baseline HBA1C	-0.365	< 0.001*
Baseline Triglycerides	0.051	0.616
Baseline HDL	0.07	0495
Baseline LDL	-0.11	0.283

At the 1-year follow-up, there was a statistically significant reduction in weight (*p* < 0.001), BMI (*p* < 0.001), WC (*p* < 0.001), fasting glucose levels (*p* < 0.001), HbA1C levels (*p* < 0.001), and triglycerides (*p* < 0.001), and a statistically significant increase in HDL (*p* < 0.001) and IGF-1 (*p* < 0.001) (Table 1).

As for the obesity-associated medical disorders, complete resolution was shown in 47/56 patients with dyslipidemia (83.9%), 28/41 patients with hypertension (68.3%), and 15/32 patients with T2DM diabetes mellitus (46.9%). There was an improvement in the remaining patients with dyslipidemia (9/56; 16.1%), 6/41 patients with hypertension (14.6%), and 12/32 patients with T2DM (37.5%) (Table 1).

The 1-year weight loss was suboptimal (%TWL < 20%) in 14 patients (14%). Comparison of the patients’ baseline characteristics according to the 1-year weight loss showed that patients with suboptimal initial clinical response (%TWL < 20%) had significantly older age (*p* < 0.001), higher fasting serum glucose levels (*p* = 0.005), and lower IGF-1 levels (*p* < 0.001) (Table 3).

**Table 3:** Comparison between the study patients according to the 1-year optimal initial clinical response

Parameter	Patients with suboptimal initial clinical response (suboptimal %TWL) (n = 14): Mean ± SD	Patients with optimal initial clinical response (optimal %TWL) (n = 86): Mean ± SD	*P*-value
Age	50.00 ± 5.519	34.86 ± 8.969	< 0.001*
Baseline weight	125.57 ± 15.068	137.84 ± 23.507	0.062
Baseline BMI	46.529 ± 4.8284	50.188 ± 8.4538	0.119
Baseline HBA1C	7.6000 ± 2.70896	6.1377 ± 1.31403	0.067
Baseline IGF-1	20.971 ± 1.4052	44.651 ± 8.9624	< 0.001*
Baseline fasting Glucose	186.14 ± 80.072	114.77 ± 33.922	0.005*
Baseline WC	105.43 ± 8.715	112.65 ± 23.854	0.267
Baseline triglycerides	157.00 ± 55.417	142.02 ± 57.039	0.364
Baseline HDL	43.00 ± 15.462	43.45 ± 9.675	0.883
Baseline LDL	133.86 ± 42.031	122.95 ± 45.154	0.401
1-year weight	102.86 ± 11.030	88.02 ± 19.600	< 0.001*
1- year BMI	37.143 ± 2.6270	32.130 ± 6.9698	< 0.001*
1-year IGF1	56.86 ± 10.805	117.07 ± 30.040	< 0.001*
1-year WC	94.43 ± 8.838	88.47 ± 23.068	0.088
1-year HBA1C	6.114 ± 1.2495	5.102 ± 0.5798	0.01*
1-year fasting glucose	142.33 ± 45.097	93.13 ± 11.408	0.003*
1-year triglycerides	142.17 ± 41.728	106.40 ± 44.011	0.01*
1-year HDL	55.83 ± 16.803	54.15 ± 9.388	0.74
1-year LDL	127.17 ± 37.651	117.83 ± 35.170	0.397

Comparison of the 1-year measures revealed that patients with suboptimal initial clinical response significantly higher weight and BMI (*p* < 0.001), fasting serum glucose levels (*p* = 0.003), HbA1C (*p* = 0.01), and triglycerides (*p* = 0.01), and significantly lower IGF-1 levels (*p* < 0.001) (Table 3).

The mean ∆ IGF-1 was 67.43 ± 25.88 ng/ml. there was statistically significant positive correlation between ∆ IGF-1 and %TWL (*r* = 0.716, *p* < 0.001) as well as ∆ IGF-1 and ∆ BMI (*r* = 0.211, *p* = 0.035).

Multiple regression analysis revealed that, among the patients baseline characteristics, the significant predictors for %TWL were the patients’ BMI (*p* < 0.001) and IGF-1 levels (*p* < 0.001). The effect of these parameters on %TWL was independent of the other patients’ parameters that were taken into account in the analysis (Table 4).

**Table 4:** Multiple regression for the prediction of the 1-year %TWL

	Unstandardized Coefficients		Standardized Coefficients	t	Sig	95.0% Confidence Interval for B	
	B	SE	Beta			Lower Bound	Upper Bound
(Constant)	-7.904	5.381		-1.469	0.145	-18.594	2.786
Age	0.026	0.045	0.029	0.575	0.567	-0.063	0.114
BMI	0.182	0.048	0.171	3.799	< 0.001*	0.087	0.278
HBA1C	0.185	0.246	0.036	0.751	0.455	-0.305	0.675
IGF-1	0.718	0.036	0.971	20.013	< 0.001*	0.647	0.790
Triglycerides	-0.002	0.008	-0.013	-0.260	0.795	-0.017	0.013
HDL	0.016	0.041	0.020	0.391	0.697	-0.065	0.097
LDL	0.012	0.009	0.062	1.400	0.165	-0.005	0.029

Testing the correlation of %TWL with the 1-year follow-up characteristics revealed that only the IGF-1 levels showed a statistically significant positive association (*p* < 0.001).

**Fig. 1:**
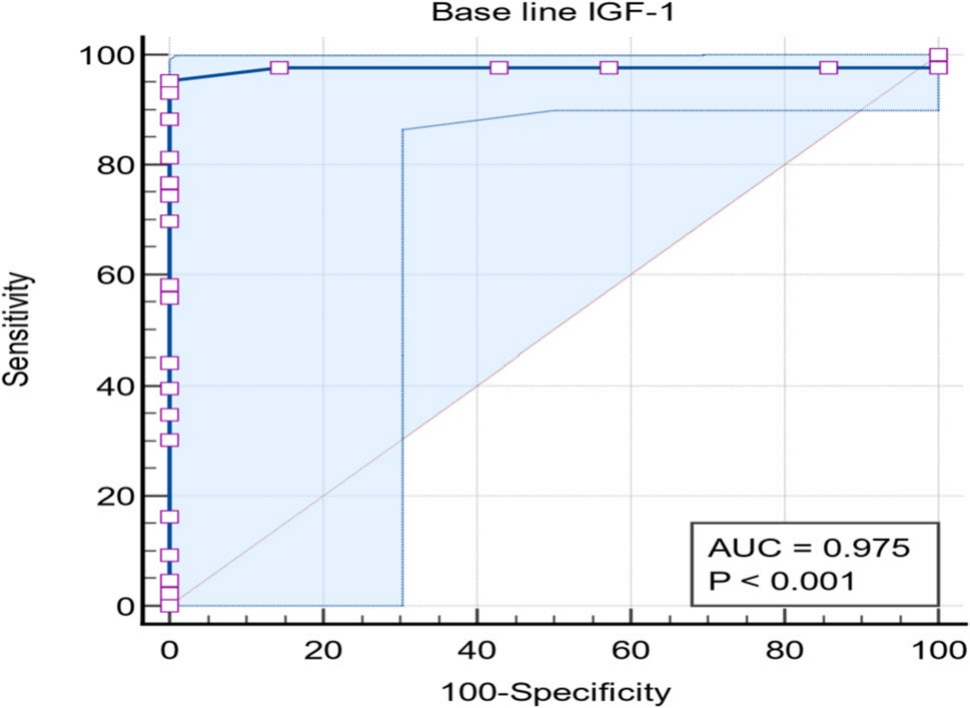
ROC curve of the diagnostic performance of IGF-1 for the prediction of postoperative suboptimal initial clinical response

ROC curve analysis to determine the discriminant ability of the baseline IGF-1 in determining optimal 1-year weight loss showed that an IGF1 cutoff value of ≤ 23 ng/ml had excellent discriminant power to detect suboptimal initial clinical response, with an AUC of 0.975, a sensitivity of 95.35%, and a specificity of 100% (Fig. 1).

## Discussion

Sleeve gastrectomy is a well-established bariatric procedure for patients with obesity who cannot achieve sustained weight loss with lifestyle modification or medical treatment [31–33]. The effect of SG is not only through the restriction of stomach volume, but other endocrinological pathways have been proposed to be a main interplay factor in the process of weight loss and the improvement of obesity complications [34]. The role of IGF-1 as an important metabolic regulator that reflects the levels of GH and partially mediates its growth effects has currently emerged [13, 35–38].

In this study, we investigated the SG effect on the metabolic profile of one hundred Egyptian adult female patients with obesity and the potential role of IGF-1. We selected female patients to provide a homogeneous study population, allowing us to minimize the impact of gender-related variations on IGF-1 levels. While IGF-1 is a crucial growth factor that plays diverse roles in both males and females, existing literature suggests that its circulating levels can vary between the sexes. Sex hormones, such as estrogen and testosterone, are known to influence IGF-1 production and regulation. Females tend to have different hormonal profiles than males, and these hormonal differences can contribute to variations in IGF-1 levels [39–41].

By focusing exclusively on female patients, we aimed to create a more homogenous study population, reducing the confounding effects of gender-related hormonal fluctuations on IGF-1 measurements. This approach enhances the internal validity of our findings and allows for a more focused investigation into the specific relationship between IGF-1 levels and weight loss outcomes in female patients undergoing sleeve gastrectomy.

The promising outcomes of LSG on weight loss and metabolic disorders are currently evidenced [31–33]. Our study emphasized this in terms of significant improvement in the lipid profile and glycemic control with high rates of resolution of associated medical disorders, besides the significant weight loss. This is supported by several previous studies that demonstrated a meaningful loss of weight and resolution of obesity-associated obesity complications after SG [31–33, 42, 43].

The SG-associated improvement of the metabolic parameters could be explained by the effect of reduced gastric volume on the amount of ingested food and the subsequent weight loss that was evidenced to affect the patients’ biochemical and metabolic parameters, including glucose levels and lipid profile [44]. However, other hormonal changes may also share the metabolic effect after LSG [36, 37, 45].

Among the possible explanations, IGF-1 might have an eminent role. Both insulin and IGF-1 have hypoglycemic and anabolic effects achieved by binding to the IGF-1 receptor and/or the insulin receptor [46], giving IGF-1 its own metabolic actions in insulin sensitivity, lipolysis, and proteolysis regulation as part of the IGF-1/insulin system [47]. Additionally, through IGF-1-mediated GH effects, the lipolysis process is stimulated by the free fatty acids released from fatty tissue, most prominently visceral fat. Moreover, hepatic triglyceride storage is maintained, and their uptake by skeletal muscles is stimulated [48]. It has been described that “fine tuning” of the IGF-1 signaling cascade is critical for proper adipogenesis [47].

IGF-1 has shown variable levels in patients with obesity, as previously described [13, 36, 49, 50]. In this study, despite being within the normal range, the preoperative IGF-1 levels were negatively correlated with the baseline WC, fasting glucose, and HbA1C levels. This elucidates the impact of abdominal obesity on IGF-1 levels and the subsequent disruption in glycemic control. The IGF-1 levels were also negatively correlated with the patients’ age. This correlation was previously described [51, 52] and explained by the reduction of GH levels in older individuals being nearly negligible in individuals aged above 60 years [52], with a subsequent decline in the hepatic production of IGF-1, which is regulated by GH.

In the present work, there was an evident elevation in the IGF-1 levels at the post-surgery follow-up. This is consistent with previous studies that reported a significant elevation in IGF-1 levels after bariatric surgery [13, 15, 35, 38, 53]. Interestingly, Mittempergher et al. [54] reported that IGF-1 levels did not exhibit significant elevation after bypass procedures, despite being significantly increased after SG. This was explained by the bypass procedure-associated deficiency in nutrients, including protein, which is needed for the improvement in IGF-1 levels after surgery [15]. However, Mittempergher et al. [54] found no significant association between baseline IGF-1 and postoperative weight loss.

Variable predictors for post-SG weight loss were described among studies [8–14]. Scarce conflicting evidence is available concerning the impact of IGF-1 levels on weight loss after SG [13, 54]. In this study, there was a significant association between ∆ IGF-1 and %TWL, pointing to the potential role of IGF-1 dynamics in influencing overall weight reduction. Moreover, after adjusting for the other confounders, BMI and IGF-1 were the baseline predictors for achieving optimal initial clinical response. This result implies the significant role of IGF-1 in the weight-loss process.

In line with our study, Ohira et al. [13] found that IGF-1 levels were a predictor for post-bariatric weight loss. Within the same context, Savastano et al. [55] found that the percentage of excess weight loss after surgery was significantly lower in patients with subnormal levels of IGF-1. The enhancing role of IGF-1 for weight loss after surgery could be explained by its anabolic role, which increases the mass of muscles, enhances the expenditure of energy, stimulates lipolysis, and regulates insulin sensitivity [15, 56]. It is worth noting that the current study showed a positive correlation of GLP-1 levels with the WC, reflecting the GH status. This finding could align with the described finding that GH causes a significant reduction in the amount of subcutaneous and visceral fat when it is used for the treatment of abdominal obesity [57].

Similar to our findings, researchers in previous studies have observed an obvious positive correlation between the baseline BMI values and the post-bariatric surgery weight loss [58–60].

While our results demonstrated a significant correlation between IGF-1 levels and various health parameters, including weight loss, it is crucial to consider the multifaceted nature of this relationship. It is imperative to recognize that correlation does not imply causation. The role of IGF-1 in metabolic regulation is complex and influenced by a myriad of factors, including nutritional status, hormonal balance, and physical activity levels [61]. Therefore, while our data suggest that IGF-1 levels may serve as a valuable biomarker in the context of weight loss, we cannot conclusively determine whether these levels are a direct causal factor, a mere indicator, or a consequence of weight loss and associated metabolic changes.

Furthermore, it is important to consider that patients with better weight loss outcomes often engage in more physical activity and have healthier dietary habits, which in turn could positively affect IGF-1 levels. This interplay highlights the importance of a holistic approach to understanding and interpreting the relationship between IGF-1 levels and weight loss. We acknowledge the need for further research to unravel the exact nature of this relationship. Future studies should aim to dissect the causal pathways and investigate how modifications in lifestyle factors could mediate the effects of IGF-1 on weight loss.

Investigating a cohort consisting exclusively of females, despite its potential limitation in generalizability, was a deliberate choice aimed at examining a more homogenous population. This approach allows for a focused analysis, minimizing the influence of gender-related variables that could confound the results. Another limitation of our study is the short-term follow-up period. Larger-scale studies with long-term follow-up are warranted to validate the clinical utility of baseline IGF-1 levels as a reliable marker for optimal initial clinical response. Furthermore, exploring the effectiveness of GLP-1 agonists in individuals with IGF-1 levels below a defined threshold presents an avenue for enhancing surgical outcomes.

## Conclusion

This study underscores the significant impact of LSG on weight loss and metabolic improvements in female patients. Notably, baseline IGF-1 levels emerged as a crucial predictor for optimal initial clinical response, emphasizing their potential as a valuable marker in guiding clinical decisions and predicting surgical outcomes in this cohort. However, it is crucial to acknowledge that our results should be interpreted with caution due to the inherent limitations of our study. The association between IGF-1 levels and weight loss outcomes, while compelling, warrants further investigation to fully understand its nature and implications.

## Data Availability

The datasets analyzed during the current study are available upon an editorial request.
